# Identifying innovations produced by primary health care centers and evaluating their scalability: the SPRINT Occitanie cross-sectional study in France

**DOI:** 10.1186/s12913-024-11237-z

**Published:** 2024-07-17

**Authors:** Alexis Vandeventer, Grégoire Mercier, Christophe Bonnel, Joana Pissarra, Grégory Ninot, François Carbonnel

**Affiliations:** 1 IDESP, Univ Montpellier, INSERM, Maison de santé pluriprofessionnelle universitaire Avicenne, 2 rue Ibn Sinaï dit Avicenne 66330 Cabestany, Montpellier, France; 2 University Department of General Practice, Faculty of Medicine of Montpellier-Nîmes, University of Montpellier, Montpellier, France; 3 CHRU Montpellier - Centre Hospitalier Régional Universitaire, Univ Montpellier, Montpellier, France; 4 Clinical Research and Epidemiology Unit, CHU Montpellier, Univ Montpellier, Montpellier, France; 5 Maison de santé pluriprofessionnelle universitaire Avicenne, 2 rue Ibn Sinaï dit Avicenne, Cabestany, 66330 France

**Keywords:** Cross-sectional studies, Diffusion of innovation, Health promotion / organization & administration, Health promotion / standards / humans, Primary Health Care / organization & administration, Process assessment, Health Care / methods, Program development, Surveys and questionnaires, Multi-professional Healthcare center MHC

## Abstract

**Background:**

Practice-based research is one of the levers identified by the World Health Organization (WHO) to strengthen primary health care. The scaling of health and social care innovations has the potential to reduce inequities in health and to expand the benefits of effective innovations. It is now rapidly gaining the attention of decision-makers in health and social care, particularly in high-income countries.

To meet the challenge of declining numbers of primary care physicians in France, Multi-professional Healthcare Centers (MHC) were created to bring together medical and paramedical professionals. They are a source of innovation in meeting the health challenges facing our populations.

Specific methodology exists to identify health innovations and assess their scalability. A working group, including end-users and specialists, has adapted this methodology to the French context and the University department of general practice of Montpellier-Nîmes (France) launched a pilot study in Occitanie, a French region.

**Objective:**

To identify and evaluate the scalability of innovations produced in pluri-professional healthcare centers in the Occitanie region.

**Methods:**

A pilot, observational, cross-sectional study was carried out. The SPRINT Occitanie study was based on a questionnaire with two sections: MHC information and the modified Innovation Scalability Self-Administered Questionnaire (ISSaQ), version 2020. The study population was all 279 MHC in the Occitanie region.

**Results:**

19.3% (54) of MHC in the Occitanie region, responded fully or incompletely to the questionnaire. Four out of 5 U-MHCs were represented. Five MHC presented multiple innovations. The average per MHC was 1.94 (± 2.4) innovations. 26% of them (*n* = 9) had high scalability, 34% (*n* = 12) medium scalability and 40% (*n* = 14) low scalability. The main innovation represented (86%) were healthcare program, service, and tool.

**Conclusions:**

In our cross-sectional study, a quarter of the innovations were highly scalable. We were able to demonstrate the importance of MHC teams in working on primary care research through the prism of innovations. Primary-care innovations must be detected, evaluated, and extracted to improve their impact on their healthcare system.

**Supplementary Information:**

The online version contains supplementary material available at 10.1186/s12913-024-11237-z.

## Background

Primary health care-oriented health systems are organized with the goal to provide the highest attainable level of outpatient healthcare services, while maximizing equity and solidarity [1]. Primary health care-oriented research is one of the 10 operational levers identified by the World Health Organization (WHO) to strengthen them [2]. The scaling of health and social care innovations has the potential to reduce inequities in health and to expand the benefits of effective innovations [3–5]. WHO defines health innovations as a new or improved solution with the transformative ability to accelerate positive health impact [6]. They are now rapidly gaining the attention of decision-makers, particularly in high-income countries [7]. They are faced with complex health and social care systems, aging populations and limited financial and human resources, justifying the need to prioritize effective scaling of beneficial innovations [8–11].

The number of French primary care doctors has fallen by 8% between 2012 and 2022 [12]. Nearly 11% of French people over the age of 17 did not have a general practitioner in 2022, and 30% lived in a "medical desert" [13, 14]. The crisis in access to healthcare is a major concern for the French population and other high-income countries [15]. The healthcare system must be updated to include organizational innovations [16]. To meet these challenges, the French government enabled the creation of multi-professional health care centers (MHCs) from 2007. An MHC is made up of at least 2 general practitioners and at least one health auxiliary such as a pharmacist, midwife, nurse, physiotherapist, speech therapist or other regulated health profession. In 2023, 2251 MHCs were in activity throughout France [17] and the goal is to double their number by 2026 [18].

The dynamics of coordinated care in France are recent. In Quebec, it began in the 1970s with the creation of local community service centers (CLCS) where patients could consult several health professionals, in conjunction with the national social security system [19].

Canada has launched promising pilot projects that, however, were not scaled [17]. To mitigate this issue, Quebec's Sustainable Health Research Center has developed a research program aiming at identifying innovations produced by primary care facilities, classify them and evaluate their scalability [11].

We launched a pilot study to replicate their study in Occitanie, a French region, named SPRINT Occitanie (*Soins PRimaires INnovations et Territoires en Occitanie*). The original study found 25% of innovations were highly scalable (ref 11 again). Considering Quebec’s extensive experience on innovation scale-up, our research hypothesis was that PHCs produce innovations in Occitanie, but in fewer numbers and less scalable in comparison.

Nearly 83% of the region's territory is under-dense in terms of access to general practice [20]. It counted 279 MHCs in March 2023, including 5 with the “University” label [21]. This label identifies U-MHCs whose role is to coordinate between care facilities, the regional agency and the medical faculty of the nearest university, to bring together research, innovation and teaching [22]. Occitanie has three university hospital centers: Toulouse, Montpellier and Nîmes. They have organized a complete ecosystem to support innovation based on Clinical Research and Innovation Delegations, which are integrated into the university hospitals. Montpellier and Nîmes also share an innovation extractor [23]. Such ecosystems are not accessible to primary care, yet. Designing innovation extractors for primary care, in conjunction with pre-existing innovation ecosystems, could enable to better structure innovations that are more scalable and efficient, and better adapted to local needs. There is, to date, no incentive policy for evaluating and scaling up innovations for MHC. Only U-MHC are invited to do so, without allocating resources for this purpose. No study has identified healthcare innovations in MHCs in the Occitanie region, nor assessed their scalability.

The objective of this pilot study was to identify and assess the scalability of innovations produced in MHCs in the Occitanie region.

## Methods

### Study design

A pilot, observational, cross-sectional study was carried out between January and March 2023.

### Questionnaire development

Innovations were defined as what was perceived as new by MHC staff [24, 25]. To ensure harmonization, we gave respondents the WHO definition of innovation. Each person was then able to interpret the innovative or non-innovative nature of the initiatives in their MHC.

The SPRINT Occitanie study was based on a questionnaire with two sections (MHC information and the modified ISSaQ questionnaire).

It was reported as per guidelines for internet e-surveys [26] and in line with the CHERRIES checklist [27].

The first 16 questions (Additional file 1) were designed to identify the MHCs and respective innovations. These included: name of the MHC, single or multi-site nature, commune, status of the person answering the questionnaire (manager, coordinator, doctor, or other), e-mail address. It also included detailed information on the MHCs: year of creation, number of professionals, number of general practitioners, number of doctors in other specialties, number of university internship supervisors, previous responses to calls for projects (care, teaching or research), collaboration with the inter-regional grouping for clinical research and innovation (GIRCI). Specific information on innovations included were the name and abbreviation, description of the innovation, links with potential partners, communication around the project and purpose of the innovation. To characterize the type of innovation, the MHC correspondent had several modalities: program, model, approach, tool, instrument, indicator, algorithm, service, policy, practice or other, taken from the WHO's International Classification of Health Interventions [28]. This classification comprises three main axes: the target (entity on which the action was carried out), the action (act carried out by an actor on the target), and the means (processes and methods by which the action was carried out). Finally, the MHC correspondent could opt to have feedback on the scalability assessment.

The second section was adapted from the Innovation Scalability Self-Administered Questionnaire (ISSaQ), version of 2020 [11]. This questionnaire was adapted to suit the idioms of metropolitan French. The SPRINT Occitanie project team consisted of the questionnaire's end-users (teacher-researchers in general practice and MHC coordinators), a hospital specialist in innovation extraction and a public health physician methodologist. The questionnaire was tested with five MHC coordinators to check comprehension and confirm completion time. Concrete examples clarifying the questions were added following their feedback. The original questionnaire (ISSaQ) and the adapted questionnaire (modified ISSaQ) are presented in Additional file 2.

The ISSaQ assessed data availability for three dimensions (theory, impact and coverage) with 16 closed questions and 6 possible answers: “Yes, No, Not planned, Not applicable or Under evaluation”. If “yes”, the user could complete the answer by mentioning what data or elements were related to the question in free text. The *Theory* dimension included a question on the conceptual model that may or may not have informed the development of the innovation. The *Impact* dimension assessed data on six elements: acceptability, feasibility, appropriation, potential effectiveness in an experimental context, effectiveness in a real-life context, and documentation of results. For the last dimension, *Coverage*, we could have answers on the scope of the innovation, its adoption by the MHC team, fidelity in implementation, sustainability, implementation in another context, compatibility with other similar interventions, conformity with health policy guidelines in the context, and finally, the presence of data on cost-effectiveness and financial and human resource requirements.

This questionnaire was transposed onto LimeSurvey software, licensed by the University of Montpellier, to be self-administered by each MHC correspondent in Occitanie.

It was possible to complete the questionnaire for a single innovation. If the MHC wished to identify more than one, the correspondent could restart the questionnaire at the beginning.

### Population

The study population was all 279 MHCs in the Occitanie region that had received the regional health agency label. Health centers and communities of healthcare professionals were not included. In each MHC, a correspondent completed the open survey. This could be the coordinator, the manager, a medical doctor or another active member of the structure. To contact them, several e-mail reminders were sent by the university department of general practice of Montpellier-Nîmes and Toulouse to the university internship supervisor attached to them. Also, the Federation of Pluriprofessional Coordinated Practice [Fédération de l’Exercice Coordonné Pluriprofessionnel] (FECOP) contacted its members on 3 occasions, and the regional health agency of Occitanie, contacted all the MHC in the region. The FECOP is commissioned by regional health agency to support MHCs in project in the region, associated care teams in a network, offer them training and pool their innovations [29].

### Statistical analysis

After collecting the data, three members of our research team proposed a blind classification of the respondent's category. After seeing the four proposals, a consensus was made by two of the three members based on the WHO definitions. The scalability score was calculated, as stipulated *by* ISSaQ, by summing up only positive responses to the 16 scalability criteria. Responses of "No, not applicable, under evaluation or not planned" were considered null. The score obtained, a maximum of 16, was then classified into 3 categories in accordance with the proposals of Ben Charif et *al*. [30]. This hierarchical classification was made according to 3 modalities: "high" (scalability greater than or equal to 10), "low" (scalability less than or equal to 3) and “medium" for the remainder.

Descriptive statistics described demographic characteristics using frequencies with percentages for categorical variables and means and standard deviations or medians and interquartile ranges for continuous variables. Only complete responses (meeting all 16 scalability criteria) were analysed.

Statistical analyses were carried out partly using Microsoft Excel® version 2304 and RStudio® version 2023.03.1 using version R 4.2.3 with the package "stats" version 4.2.3.

## Results

### Participants

**Fig. 1:**
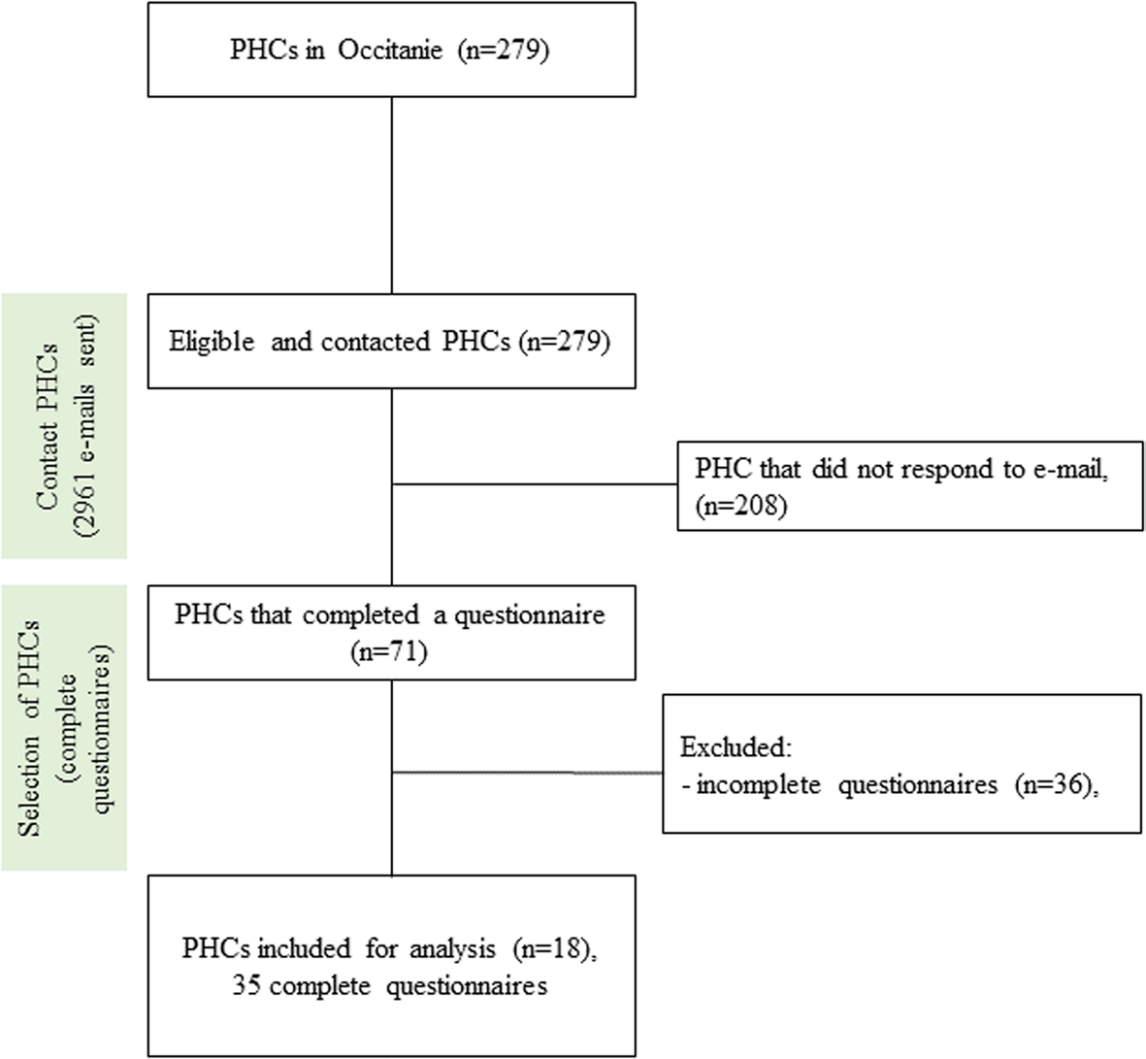
SPRINT Occitanie study flow chart

Between January and March 2023, 35 complete responses, i.e., 35 innovations, were collected from 18 different MHCs (Fig. 1).

The Checklist for Reporting Results of Internet E-Surveys (CHERRIES) is in Additional file 3.

19.3% of MHCs in the Occitanie region, i.e. 54 MHCs, responded fully or incompletely to the questionnaire. Among these, 35 questionnaires from 18 MHC were complete and analysed. Five MHCs presented multiple innovations: 11 for the U-MHC in Cabestany (Pyrénées-Orientales), 5 for the U-MHC in Vergèze (Gard), and 2 for the MHC in Mende (Lozère), the MHC in Bessèges (Gard) and the MHC in Pont-St-Esprit (Gard) (Table 1). The average per MHC was 1.94 (± 2.4) innovations. The MHC that responded incompletely were contacted to offer support in completing the questionnaire, yet none responded.

**Table 1:** Profile of MHCs responding to the SPRINT Occitanie Study

**Total number of MHCs**	18	
	**N**	**(%)**
***Department***		
Gard	5	(28)
Haute Garonne	3	(17)
Hérault	6	(33)
Lozère	2	(11)
Pyrénées-Orientales	2	(11)
***Organization***		
Multisite	16	(89)
Single-site	2	(11)
***MHC Correspondent***		
Coordinator	12	(67)
Managing Practitioner	6	(33)
***Partnerships***		
Funding calls for projects	8	(44)
Links with CHU and GIRCI	3	(16)
No partnership	7	(40)
***MHC characteristics***		
	Mean	SD
Age of MHC^b^	5	(4)
Number of Health professionals	32	(14)
Number of General Practitioners	6	(3)
Number of Other physicians	2	(3)
Number of University internship supervisors	4	(2)
Number of patients in active file	6957^a^	(3646^a^)

^a^data missing for one MHC, carried out on 17 MHCs

^b^Age in years, average calculated from the year of creation mentioned in the questionnaire response

The MHCs were recent, mainly multi-site (89%) and mostly located in Eastern Occitanie (83%) (Table 1).

### Type of innovation

**Fig. 2:**
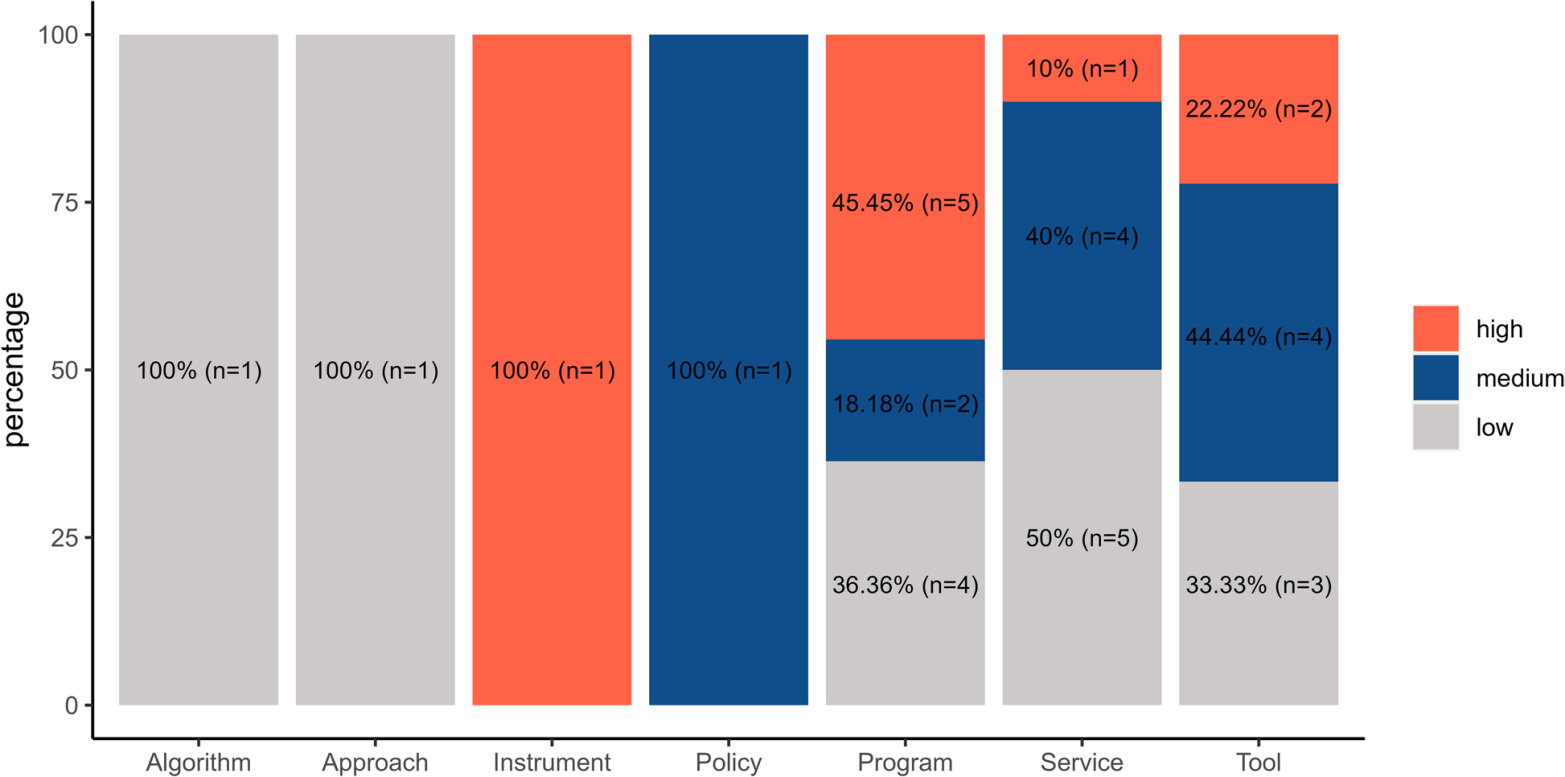
Scalability ranking by category of innovations

**Fig. 3:**
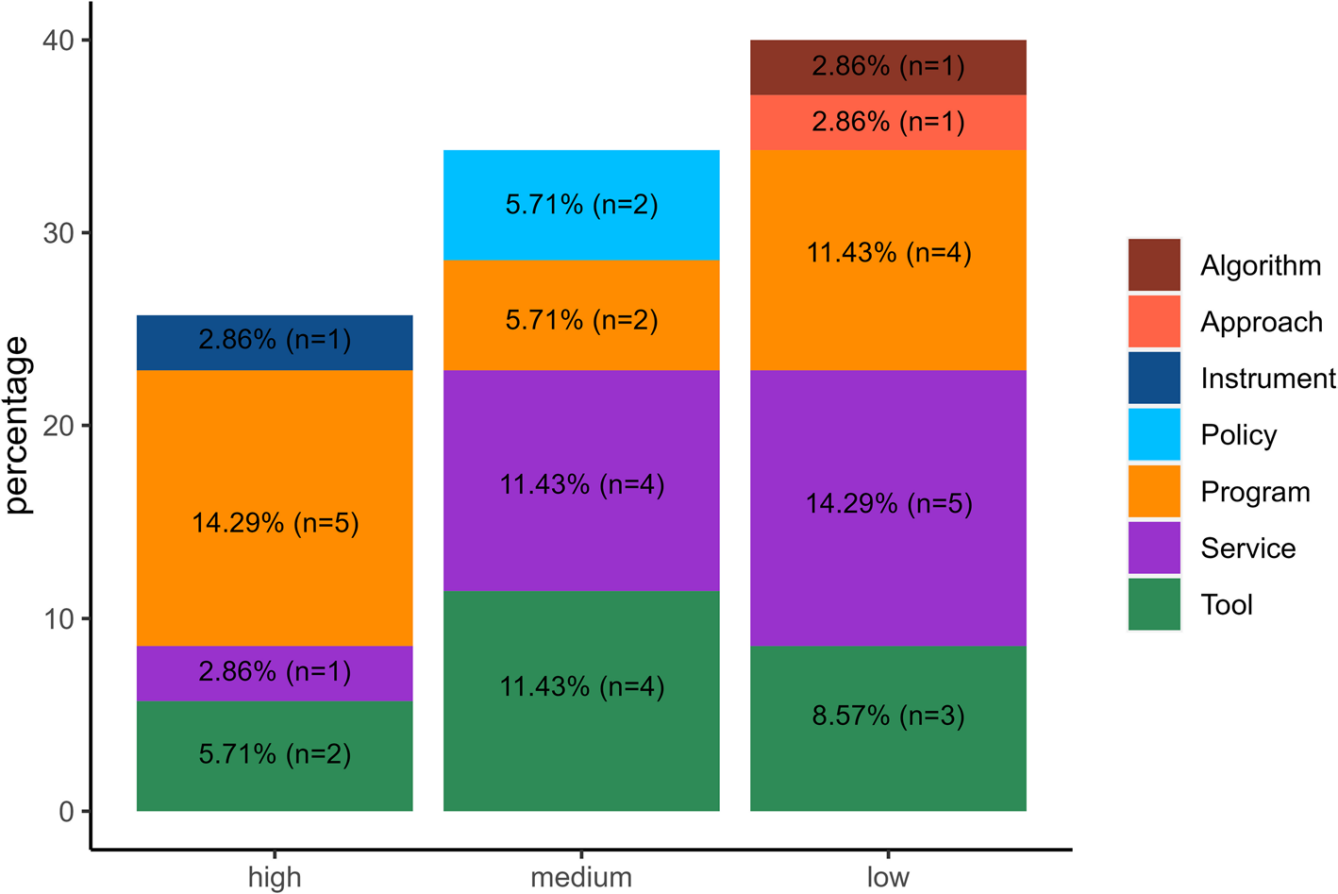
Categories of innovations by scalability ranking

Most innovations (86%) concerned a healthcare program (*n* = 11, 31%), a patient service (*n* = 10, 29%) or a tool (*n* = 9, 26%) (Fig. 2). We assessed the scalability levels of the described innovations (Figs. 2 and 3).

26% of innovations (*n* = 9) had high scalability, 34% (*n* = 12) medium scalability and 40% (*n* = 14) low scalability. Additional file 4 lists the name of the innovation and the MHC that described it, by scalability level group.

Over half of the evaluated innovations (60%) had a high or medium scalability score. Particularly, nine interventions were highly scalable, and mostly pertained to the program category (*n* = 5, 26%) (Fig. 2).

### Scalability

The results of the number of modified ISSaQ criterion to assess innovation scalability showed the average score for all innovations was 5.86 (± 3.98) (Table 2).

**Table 2:** Number and proportion of criteria evaluated, and summary of the scalability of SPRINT Occitanie innovations

	Modified ISSaQ Criterion		
TOTAL	Number of innovations	*N* = 35	
	Scalability, mean (SD)	5.86 (± 3.98)	
		Criterion Assessed	
Dimension		*N*	%
Use of theory	Innovations developed with theory	18	51
Impact assessments	Acceptability	17	49
	Feasibility	16	46
	Appropriation	8	23
	Experimental efficiency	6	17
	Real efficiency	15	43
	Documented Results	12	34
Setting and coverage assessments	Reach	15	43
	Adoption	17	49
	Fidelity	14	40
	Sustainability	12	34
	Implemented in setting comparable to target setting	7	20
	Compatibility with similar innovations in target settings	6	17
	Consistency with policy directives	23	66
Cost assessments	Cost-effectiveness	1	3
	Resources needed for the scaling up	18	51

On average, 51% of innovations had an assessment for the "theory" dimension, 35% for the "impact" dimension, 38% for the "coverage" dimension and 28% for the "cost" dimension (Table 2). Additional file 4 contains a list of all innovations, with all criteria, evaluated or not. The empirical maximum scalability score was 13, and the minimum was 0.

## Discussion

This study aimed at identifying innovations produced in MHCs in the Occitanie region, and evaluating their scalability. To this end, we replicated a previous study that reported 25% of highly scalable innovations among 24 analysed innovations, and we hypothesized lower rates of highly scalable innovations given the more recent nature of our region’s primary care coordinated networks. Our study analyzed 18 PHCs in the Occitanie region, which presented 35 innovations. We identified multiple innovations per MHC, but few MHCs are represented in the results (18 out of 279). We found the average scalability of around 6, arguably low compared with previous studies that reported an average scalability of 11 [11]. This result was expected, confirming our initial hypothesis, and several factors may explain it. The dynamics of coordinated care in France are recent, particularly when compared with Quebec.It is still difficult for MHC teams to get involved in research projects, as they are not very well structured. Encouragingly, 44% of the MHCs in the study have already received funding through calls for projects.

Between 2012 and 2017, the Ministry of Health and Prevention's objective was to label one U-MHC per department, with the aim of carrying out networked primary care research [22, 31]. We note that 4 of the 5 U-MHCs in Occitanie took part in our study, resulting in a high representation. The structuring of a network of U-MHCs is currently underway as part of the F-CRIN project, led by the supervisory ministries in conjunction with the French National Council of Teaching Generalists [32]. The GIRCIs are responsible for the coordination of regional initiatives and providing support to healthcare establishments, facilities and centers in the field of applied health research and innovation [33]. They run calls for projects, such as RESPIR (Inter-Regional Primary Care Research) since 2021. Only three MHCs indicated that they had worked with the GIRCI. It should be noted that these were the two oldest U-MHCs in the region, and the MHC where the FECOP manager works. These factors should not obscure the fact that research is at an early stage of maturity for all MHC care teams. Our results reflected this since, despite of predominantly high and medium scalability scores, there was still a substantial number of low scalability innovations, and difficulties in responding to the questionnaire criteria reported by participating teams. There is a risk of a breach of equality between the pilot structures and the others. This shows how important it is to structure a local network, clearly identified by the MHCs' correspondents, to help them design and improve their innovations. Still, these laid foundations will allow the creation of an ecosystem that benefits and reaches all. Altogether, these collective initiatives involving public institutions and university laboratories should provide medium- and long-term opportunities for the creation of health innovations and respective scaling [34].

### Strengths and limitations

The main limitation of this study stems from the study design, as cross-sectional studies imply selection bias. Some MHCs responded several times, leading to a potential desirability bias. We tried to mitigate this by taking care to contact each MHC several times, using several different email addresses senders as well as other communication channels. This was an exploratory study, not intended to be exhaustive. We kept the questionnaire open online for 3 months, to give as many MHC correspondents as possible time to respond. Ranking biases, notably memorization bias, linked to self-administration of the questionnaire, were mitigated by making each scalability criterion explicit via a concrete example that was threaded throughout the questionnaire. We also provided a contact person to help correspondents. In some MHCs, several correspondents answered the questionnaire together, but we did not quantify this, nor do we know the rate at which the questionnaire was reviewed. The ISSaQ was updated in March 2023, implying further refinement. It incorporates Likert scales and new criteria for assessing scalability have been added [35]. In our scalability analysis, each criterion has the same weight as the others, although some are more relevant to one type of innovation than others.

SPRINT Occitanie was based on a Canadian study in a different and not very comparable healthcare system.

Our success in mobilizing one-fifth of the MHCs in Occitanie is encouraging, but it also shows that most of them are still far removed from the research dimension. Most responses came from MHC with University training supervisors, in conjunction with the Montpellier-Nîmes Faculty of Medicine, which carried out the study.

### Perspectives

The exploratory work carried out by the SPRINT Occitanie study is a first step. The creation of a website, considering appropriate scalability questionnaire, could enable us to collect future innovations and build an extractor for primary care in the long term. This site could be the entry and development base for innovations in primary care. The innovation ecosystem of university hospitals and universities should be key partners. This could help creating a virtuous cycle raising questions from practice, conducting experiments, finding results, and producing evidence that can serve the purpose of improving patient care and the health of the population [36].

## Conclusions

Practice-based research supporting the development, implementation, and evaluation of innovations in primary care contributes to the improvement of patient care and the health of the population [36]. Our study showed there are promising foundations, with numerous and diverse high and medium scalability innovations in the pipeline, and a favorable ecosystem to expand this work.

### Supplementary Information

Additional file 1. Questions to identify the MHCs and the innovation described. French and English versions.

Additional file 2. Modified ISSAQ questionnaire. French and English versions.

Additional file 3. CHERRIES checklist.

Additional file 4. List of identified innovations produced by MHC, by scalability level group.

AAdditional file 5. Research Ethics Committee of the University of Montpellier approval.

## Data Availability

The data sets generated during and/or analyzed during this study are available from the corresponding author on reasonable request.

## Abbreviations

CLCS: Local community service centers
GIRCI: Groupement Inter-régional de recherche clinique et d’innovation inter-regional grouping for clinical research and innovation
ISSaQ: Innovation Scalability Self-Administered Questionnaire
MHC: Multi-professional health care center
SPRINT Occitanie: Soins PRimaires INnovations et Territoires en Occitanie (Primary Care Innovation and Territories In Occitanie)
U-MHC: University multi-professional health care center
WHO: World Health Organization
