# Supplementary material for: Identifying innovations produced by primary health care centers and evaluating their scalability: the SPRINT Occitanie cross-sectional study in France
Source: BMC Health Serv Res. 2024 Jul 17;24:824. doi: 10.1186/s12913-024-11237-z (PMC11253355; doi:10.1186/s12913-024-11237-z)
Supplement: Supplementary file 1 — Additional file 1. Questions to identify the MHCs and the innovation described. French and English versions. [file 12913_2024_11237_MOESM1_ESM.pdf]

Appendix 1 :

Questions to identify the MHC and the innovation described.

FRENCH VERSION

A. L'équipe et la MSP

1. Quel est le nom de votre MSP ?

2. Quelle est la ville de votre MSP (ou territoire de la MSP si elle est multi-site) ?

3. Est-ce que votre MSP comprend un seul site ou plusieurs ?

- Monosite
- Multisite

4. Quel est votre statut dans la structure ? (Par exemple, gérant, coordonnateur, médecin, infirmier etc.) *

5. Quelle est votre adresse e-mail ?

6. Seriez-vous d'accord pour nous transmettre le projet de santé de la MSP ?

8. Avez-vous obtenu des financements dans le cadre d'appel à projet (soins, pédagogie, recherche, …)?

Si oui, merci de préciser combien de financements :

9. Travaillez-vous en lien avec une direction de la recherche et de l'innovation d'un établissement de santé, un Groupement Interrégional pour la Recherche Clinique et l'Innovation (GIRCI) ou un extracteur d'innovation ? *

Si oui, lesquels ?

B. Renseignements généraux sur l'innovation

1. Quel est le nom de l'innovation ?

2. Quelle est l'abréviation du nom de l'innovation ?

3. Quel est le type d'innovation ?

- Programme
- Modèle
- Approche
- Outil
- Instrument
- Indicateur

- Algorithme
- Service
- Politique
- Pratique
- Autre

Si vous avez des difficultés à typer votre innovation, pas d'inquiétude, nous pourrons la reclasser selon la description que vous donnerez à la question suivante.

4. Merci de décrire votre innovation :

5. Avez-vous eu besoin de partenaires pour concevoir l'innovation ?

On entend par partenaire un établissement de santé public ou privé, une entreprise, une collectivité territoriale (mairie, département, région), une startup, une autre MSP, une CPTS, Facultés/universités, une association etc...

Si oui, lesquels ?

6. Avez-vous communiqué sur cette innovation ?

Si oui, merci de préciser :

- presse grand public
- site internet de la MSP
- article scientifique
- poster
- communication orale en congrès
- thèse
- Autre:

7. Quel est le but de l'innovation ?

ENGLISH VERSION

A. The team and the MHC

1. What is the name of your MHC?

2. What is the city of your MHC (or MHC territory if multi-site)?

3. Does your MHC comprise a single site or several?
- Single site
- Multisite

4. What is your status in the structure? (For example, manager, coordinator, doctor, nurse etc.)

5. What is your e-mail address?

6. Would you agree to send us the MHC's health project?

8. Have you obtained funding for any other projects (care, education, research, etc.)?
If yes, please specify how many financings.

9. Do you work with a research and innovation department of a healthcare establishment, a Groupement Interrégional pour la Recherche Clinique et l'Innovation (GIRCI) or an innovation extractor?
If yes, which ones?

B. General information on innovation

1. What is the name of the innovation ?

2. What is the abbreviation of the innovation's name ?

3. What is the type of innovation ?
- Program
- Model
- Approach
- Tool
- Instrument
- Indicator
- Algorithm
- Service
- Policy
- Practice
- Other

If you're having trouble typing your innovation, don't worry, we can reclassify it according to the description you give in the next question.

4. Please describe your innovation :

5. Did you need partners to develop the innovation?
By partner, we mean a public or private healthcare establishment, a company, a local authority (town hall, department, region), a start-up, another MHC, a CPTS, faculties/universities, an association etc...
If so, which ones?

6. Have you communicated on this innovation?
If so, please give details:
- general press
- MHC website
- scientific article
- poster
- conference paper
- thesis
- other:

7. What is the purpose of the innovation?
