# Supplementary material for: Identifying innovations produced by primary health care centers and evaluating their scalability: the SPRINT Occitanie cross-sectional study in France
Source: BMC Health Serv Res. 2024 Jul 17;24:824. doi: 10.1186/s12913-024-11237-z (PMC11253355; doi:10.1186/s12913-024-11237-z)
Supplement: Supplementary file 2 — Additional file 2. Modified ISSAQ questionnaire. French and English versions. [file 12913_2024_11237_MOESM2_ESM.pdf]

<u>Appendix 2</u> :

FRENCH VERSION

Questionnaire ISSaQ version adaptée par l'équipe SPRINT Occitanie

En supplément les questions originales si elles différents de la version adaptée.

1/ Une théorie ou un modèle conceptuel a-t-il éclairé l'élaboration de l'innovation ?
Exemple : un outil de sevrage tabagique créée d'après les stades du changement de Prochaska et Di Clemente)
Oui/Non/Non prévue/Sans objet/ en cours d'évaluation
Commentaire (texte long)

2/ Disposez-vous de données portant sur l'acceptabilité de l'innovation ?
On entend par acceptabilité, le fait que les patients et/ ou les professionnels de santé comprennent et acceptent de participer à l'innovation
Exemple : un outil de sevrage tabagique facilement compris par le patient et naturellement proposé par le professionnel de santé
Oui/Non/Non prévue/Sans objet/ en cours d'évaluation
Commentaire (texte long)

3/ Disposez-vous de données portant sur la faisabilité de l'innovation ?
On entend par faisabilité, la mesure selon laquelle l'innovation est compatible, pertinente et applicable dans un contexte donné
Exemple : un outil de sevrage tabagique applicable au sein de votre équipe pluriprofessionnel, pertinent pour le patient et utilisable dans le contexte de votre MSP
Oui/Non/Non prévue/Sans objet/ en cours d'évaluation
Commentaire (texte long)

4/ Disposez-vous de données portant sur l'appropriation de l'innovation ?
On entend par appropriation, la mesure selon laquelle l'innovation intègre les valeurs et croyances de la population cible.
Exemple : un outil de sevrage tabagique en lien avec la population, son besoin, ses caractéristiques intrinsèques (culture, habitudes locales de vie)
Oui/Non/Non prévue/Sans objet/ en cours d'évaluation
Commentaire (texte long)
Version originale par Ben Charif et al. :Disposez-vous de données portant sur l'appropriation de l'innovation ?

5/ Disposez-vous de données portant sur l'efficacité potentielle de l'innovation *c'est-à-dire, testée dans des conditions expérimentales* ?
Exemple : un outil de sevrage tabagique que vous avez pu tester en expérimentant avant de le proposer à vos patients
Oui/Non/Non prévue/Sans objet/ en cours d'évaluation
Commentaire (texte long)

6/ Disposez-vous de données portant sur l'efficacité de l'innovation en contexte réel, c'est-à-dire, *testée dans des conditions du monde réel* ?
Oui/Non/Non prévue/Sans objet/ en cours d'évaluation
Commentaire (texte long)

7/ Les résultats des évaluations de l'innovation ont-ils été documentés ?
Exemple : un outil de sevrage tabagique mis en place dans votre MSP avec des évaluations de la mise en place, de l'expérience vécue par le patient et de son efficacité
Oui/Non/Non prévue/Sans objet/ en cours d'évaluation
Merci de préciser : (texte long)

8/ Disposez-vous de données sur la portée de l'innovation ?
On entend par portée, la proportion de la population cible expérimentant l'innovation.
Par exemple, dans le cas d'une innovation visant 10 membres de ma MSP, si 5 l'ont expérimenté, alors la portée est de 50%.
Oui/Non/Non prévue/Sans objet/ en cours d'évaluation
Commentaire (texte long)

9/ Disposez-vous de données sur l'adoption de l'innovation ?
On entend par adoption, la proportion de contextes ou milieux qui utilisent l'innovation.
Par exemple, dans le cas d'une innovation visant 1 des 3 cabinets infirmiers de ma MSP, si 1 des cabinets l'a adopté, alors l'adoption est de 33%.
Oui/Non/Non prévue/Sans objet/ en cours d'évaluation
Commentaire (texte long)

10/ Disposez-vous de données sur la fidélité de la mise en œuvre et l'utilisation de l'innovation ?
On entend par fidélité, la mesure selon laquelle l'innovation est mise en œuvre et utilisée comme prévu.
Oui/Non/Non prévue/Sans objet/ en cours d'évaluation
Commentaire (texte long)

11/ Disposez-vous de données sur la pérennisation de l'innovation ?
On entend par pérennisation, la mesure selon laquelle l'innovation est maintenue au fil du temps.
Oui/Non/Non prévue/Sans objet/ en cours d'évaluation
Commentaire (texte long)

12/ Avez-vous implémenté l'innovation dans un contexte comparable à celui dans lequel elle a été créée *c'est-à-dire au niveau individuel, de la communauté, culture, politique, du personnel ou de l'organisatio*n ?
Par exemple, vous avez créé une innovation au sein de votre MSP que vous avez déployé dans votre CPTS.
Oui/Non/Non prévue/Sans objet/ en cours d'évaluation
Commentaire (texte long)
Version originale de Ben Charif et al. : Avez-vous mis en œuvre l'innovation dans un contexte (p. ex, au niveau individuel, de la communauté, culturel, politique, du personnel ou de l'organisation) comparable au nouveau contexte dans lequel elle sera mise à l'échelle ?

13/ Avez-vous évalué si l'innovation est compatible avec des interventions similaires dans le même contexte ?
Exemple : un outil de sevrage tabagique développé dans votre MSP compatible avec d'autres interventions de sevrage proposé par un établissement public de santé à proximité, des outils de la CPAM etc..
Oui/Non/Non prévue/Sans objet/ en cours d'évaluation
Commentaire (texte long)

14/ Avez-vous déterminé si l'innovation est conforme aux directives en matière de politique du contexte dans lequel elle sera déployée ? *Par exemple, vous avez mis en place une innovation pour votre MSP, compatible avec les directives du projet régional de santé et les recommandations de la Haute Autorité de Santé.*
Oui/Non/Non prévue/Sans objet/ en cours d'évaluation
Lesquels ? Commentaire (texte long)
Version originale de Ben Charif et al. : Avez-vous déterminé si l'innovation est conforme aux directives en matière e politique du contexte dans lequel l'innovation sera mise à l'échelle ?

15/ Disposez-vous de données sur le rapport coûts-efficacité de l'innovation *c'est à dire comparativement à des innovations équivalentes existantes ou des innovations alternatives* ?
Oui/Non/Non prévue/Sans objet/ en cours d'évaluation
Commentaire (texte long)

16/ Disposez-vous de données portant sur les ressources financières et humaines nécessaires pour mettre à l'échelle l'innovation ?
On entend par mettre à l'échelle le fait de déployer l'innovation sur un autre territoire différent du vôtre.
Oui/Non/Non prévue/Sans objet/ en cours d'évaluation
Commentaire (texte long)

ENGLISH VERSION

ISSaQ questionnaire adapted by the SPRINT Occitanie team

Supplement original questions if different from adapted version.

1/ Did a theory or conceptual model inform the development of the innovation?
Example: a smoking cessation tool created according to Prochaska and Di Clemente's stages of change)
Yes/No/Not planned/Not applicable/under evaluation
Comment (long text)

2/ Do you have data on innovation acceptability?
Acceptability means that patients and/or healthcare professionals understand and agree to participate in the innovation.
Example: a smoking cessation tool that is easily understood by patients and naturally suggested by healthcare professionals.
Yes/No/Not planned/Not applicable/under evaluation
Comment (long text)

3/ Do you have data on the feasibility of the innovation?
By feasibility, we mean the extent to which the innovation is compatible, relevant and applicable in a given context.
Example: a smoking cessation tool applicable within your multi-professional team, relevant for the patient and usable in the context of your MHC.
Yes/No/Not planned/Not applicable/under evaluation
Comment (long text)

4/ Do you have data on innovation appropriation?
By appropriation, we mean the extent to which the innovation integrates the values and beliefs of the target population.
Example: a smoking cessation tool in line with the population, its needs and intrinsic characteristics (culture, local lifestyle habits).
Yes/No/Not planned/Not applicable/under evaluation
Comment (long text)
Ben Charif et al. Original version: Do you have data on the appropriation of the innovation?

5/ Do you have data on the potential effectiveness of the innovation, i.e., tested under experimental conditions?
Example: a smoking cessation tool that you could test by experimenting before offering it to your patients.
Yes/No/Not planned/Not applicable/under evaluation
Comment (long text)

6/ Do you have data on the effectiveness of the innovation in a real context, i.e., tested under real-world conditions?

Yes/No/Not planned/Not applicable/under evaluation
Comment (long text)

7/ Have the results of innovation evaluations been documented?
Example: a smoking cessation tool implemented in your MHC with evaluations of implementation, patient experience and effectiveness.
Yes/No/Not planned/Not applicable/under evaluation
Please specify: (long text)

8/ Do you have data on the innovation's reach?
By reach, we mean the proportion of the target population experimenting with the innovation. For example, in the case of an innovation targeting 10 members of my MSP, if 5 have experienced it, then the reach is 50%.
Yes/No/Not planned/Not applicable/under evaluation
Comment (long text)

9/ Do you have data on innovation adoption?
By adoption, we mean the proportion of contexts or environments that use the innovation. For example, in the case of an innovation targeting 1 of the 3 nursing practices in my MSP, if 1 of the practices has adopted it, then adoption is 33%.
Yes/No/Not planned/Not applicable/under evaluation
Comment (long text)

10/ Do you have data on the fidelity of innovation implementation and use?
Fidelity refers to the extent to which the innovation is implemented and used as intended.
Yes/No/Not planned/Not applicable/under evaluation
Comment (long text)

11/ Do you have data on the sustainability of the innovation?
Sustainability refers to the extent to which the innovation is maintained over time.
Yes/No/Not planned/Not applicable/under evaluation
Comment (long text)

12/ Have you implemented the innovation in a context comparable to the one in which it was created, i.e. at individual, community, cultural, political, staff or organizational level?
For example, you created an innovation within your MSP that you deployed in your CPTS.
Yes/No/Not planned/Not applicable/under evaluation
Comment (long text)
Ben Charif et al. Original version: Have you implemented the innovation in a context (e.g. individual, community, cultural, political, staff or organizational) comparable to the new context in which it will be scaled up?

13/ Have you assessed whether the innovation is compatible with similar interventions in the same context?

Example: a smoking cessation tool developed in your MSP compatible with other cessation interventions offered by a nearby public health institution, CPAM tools, etc.?
Yes/No/Not planned/Not applicable/under evaluation
Comment (long text)

14/ Have you determined whether the innovation complies with policy guidelines for the context in which it will be deployed?
For example, you have implemented an innovation for your MSP that is compatible with the guidelines of the regional health project and the recommendations of the Haute Autorité de Santé.
Yes/No/Not planned/Not applicable/under evaluation
Which innovations? Comment (long text)
Ben Charif et al. Original version: Have you determined whether the innovation complies with the policy guidelines for the context in which it will be scaled up?

15/ Do you have data on the cost-effectiveness of the innovation, i.e. compared with equivalent existing innovations or alternative innovations?
Yes/No/Not planned/Not applicable/under evaluation
Comment (long text)

16/ Do you have data on the financial and human resources required to scale up the innovation?
Scaling up means deploying the innovation in a different territory from your own.
Yes/No/Not planned/Not applicable/under evaluation
Comment (long text)
