# Supplementary material for: Identifying innovations produced by primary health care centers and evaluating their scalability: the SPRINT Occitanie cross-sectional study in France
Source: BMC Health Serv Res. 2024 Jul 17;24:824. doi: 10.1186/s12913-024-11237-z (PMC11253355; doi:10.1186/s12913-024-11237-z)
Supplement: Supplementary file 5 — AAdditional file 5. Research Ethics Committee of the University of Montpellier approval. [file 12913_2024_11237_MOESM5_ESM.pdf]

UNIVERSITÉ DE MONTPELLIER

Direction de la Recherche et des Études Doctorales
Bureau des instances et d'appui réglementaire
Secrétariat du Comité d'Ethique de la Recherche
dred-saisine-cer@umontpellier.fr
04.67.14.30.23

CC 404 - Place Eugène Bataillon
34095 Montpellier Cedex 5

WWW.UMONTPELLIER.FR

Comité d'Éthique de la Recherche de l'Université de Montpellier

Avis consultatif n° UM 2022-013-bis

Vu Règlement (UE) 2016/679 du Parlement européen et du Conseil du 27 avril 2016 relatif à la protection des personnes physiques à l'égard du traitement des données à caractère personnel et à la libre circulation de ces données, et abrogeant la directive 95/46/CE,

Vu le Règlement Intérieur du Comité d'Éthique de la Recherche de l'Université de la Recherche,

Les membres du Comité d'Éthique de la Recherche de l'UM entendus en sa séance du 31 janvier 2023.

Le Comité d'Éthique de la Recherche de l'Université de Montpellier rend un avis favorable sur le projet « Projet SPRINT (Soins Primaires Innovations Territoires) » soumis par Dr. François Carbonnel et M. Alexis Vandeventer.

Fait à Montpellier

Le 17/02/2023

Le Président du Comité d'Éthique de la Recherche
de l'Université de Montpellier

Thierry Lavabre-Bertrand
